# The Top 100 Most Cited Articles on Musculoskeletal Radiology: A Bibliometric Analysis

**DOI:** 10.7759/cureus.74137

**Published:** 2024-11-21

**Authors:** Lucy Moore, Hannah Hughes, Eric Heffernan

**Affiliations:** 1 Radiology, St. Vincent's University Hospital, Dublin, IRL

**Keywords:** computed tomography scan, interventional radiology, mri, msk radiology, musculoskeletal disorders, musculoskeletal pain, musculoskeletal radiology, ultrasound

## Abstract

The number of citations an article receives is reflective of its impact on the scientific community. The top 100 most cited articles were identified using the Web of Science database. Data relating to the publication year, publishing journal, number of citations, primary institution, journal impact factor, authorship, country of origin, radiological modality, and keywords were collected. In the top 100 list, the number of citations per article ranged from 149 to 709 (median 208; mean 240). Per article, the average number of citations per year ranged from five to 60 (median 12; mean 26). The United States was the most common country of origin (n=74). The journal with the greatest number of articles was Radiology (n=34). The University of California contributed the most articles (n=11). This study presents a detailed analysis of the top 100 most cited articles published in musculoskeletal radiology. It affords clinicians and researchers an understanding of the characteristics of the current most influential research papers in this field. It also highlights research trends and areas that may benefit from further research.

## Introduction and background

Bibliometric analysis (BA) is a quantitative analysis method that assesses the quality and impact of scholarly work in a process that involves examining published literature on a specific subject [1,2]. One means of analysis is citation frequency, which evaluates how frequently a publication is cited by other researchers [3]. This analysis is based on the premise that if an academic publication is cited highly, it has likely had a significant influence on its particular field of interest. BA enables us to determine research trends, indicates educational interest in a subject, or highlights potential educational gaps [3].

Musculoskeletal disorders (MSDs), consisting of over 150 diagnoses, are broadly defined as diseases or injuries of osseous structures, muscles, joints, tendons, ligaments, supportive soft tissues, and cartilage [4]. Musculoskeletal (MSK) radiology is a subspecialty of radiology that has evolved significantly in the time since the first X-ray was used to diagnose a fracture. Its scope is wide, from the radiographs used to diagnose MSDs to minimally invasive procedures that effectively target and treat these disorders [5]. The ability of the MSK radiologist to use image-guided therapeutics and diagnostics, for example, radiofrequency ablation and ultrasound-guided injections or biopsies, has meant increased patient comfort, earlier diagnosis, and financial savings [5].

Our study aims to provide a detailed and comprehensive analysis of the most influential articles pertaining to MSK radiology, thus providing insights into the subspecialty and aiming to direct researchers towards areas where there is a need for increased research and funding activities. 

## Review

Materials and methods

A retrospective BA of the most highly cited articles in MSK radiology was conducted in June 2024. The data presented therefore reflect the citation counts at that time. 

Articles were found using the Web of Science (WoS, Clarivate Analytics, Philadelphia, Pennsylvania, United States) citation indexing service. This service uses the database Medline (US National Library of Medicine, Bethesda, Maryland, United States). Journal impact factors (IF) were derived from the Clarivate "Journal Citation Reports" 2023 database [6].

The following search terms were used: "musculoskeletal AND radiology", OR "musculoskeletal AND (computed tomography OR CT)" OR "musculoskeletal AND (magnetic resonance OR MR)" OR "musculoskeletal AND (ultrasound OR sonography OR US)" OR "musculoskeletal AND (X ray OR radiograph OR XR)" OR "musculoskeletal AND (interventional radiology OR IR)". The search was refined by the WoS categories "Radiology, Nuclear Medicine, Medical Imaging". There were no limitations placed on language or year of publication. 

Journal articles were sorted by the number of citations from the most cited to the least cited. Articles were included if they were published in a radiology-specific journal and focused on diagnostic imaging interpretation, imaging technique, comparison of modalities, utility and role of different imaging modalities, or trends in MSK radiology. Articles were excluded if they did not pertain to the MSK system or radiology. The full text of each article was reviewed to identify the primary focus of the study. 

Two reviewers (LM and HH) evaluated each article and gathered data relating to publication year, publishing journal, number of citations, primary institution, authorship, country of origin, journal IF, radiological modality, and keywords. Author institutions and country of origin were derived from author affiliations. 

Results

The most cited article had 709 citations, while the least cited article had 149 citations. The mean number of citations per article was 240 with a median of 208 (range: 709-149). The mean number of citations per article per year was 26, with a median of 12 (range: 60-65). The article with the most citations was "Femoroacetabular impingement: radiographic diagnosis - what the radiologist should know" published in the American Journal of Roentgenology in 2007, with 709 citations [7]. When ranked by citations per year, the top article was "Shear-wave elastography: basic physics and musculoskeletal applications" published in Radiographics in 2017 [8]. The top 50 most cited articles relating to MSK radiology are listed in order of citation count in Table 1. 

**Table 1:** Characteristics of the top 100 most cited articles in musculoskeletal radiology No: number; Cit: citations; Pub: published; MRI: magnetic resonance imaging; MR: magnetic resonance; Gd(DTPA): gadolinium-labeled diethylenetriaminepentaacetic acid; CT: computed tomography; AFIP: Armed Forces Institute of Pathology; 4T: 4 tesla; 2D U-Net: two-dimensional U-NET; NMR: nuclear magnetic resonance; IEEE: Institute of Electrical and Electronics Engineers; 3T: 3 tesla; FDG PET: 18-fluro-deoxyglucose positron emission tomography; 3D: three-dimensional; TE: time to echo; UTE: ultrashort time to echo; OA: osteoarthritis; SAPHO: synovitis, acne, pustulosis, hyperostosis, and osteitis; SPECT: single-photon emission computed tomography

Rank	No. of cit.	Cit. per year	Year pub.	Journal	Article title
1	709	42	2007	Radiology	Femoroacetabular impingement: radiographic diagnosis - what the radiologist should know
2	533	21	1999	Magnetic Resonance in Medicine	Nondestructive imaging of human cartilage glycosaminoglycan concentration by MRI
3	510	26	2004	Seminars in Musculoskeletal Radiology	Cartilage MRI T2 relaxation time mapping: overview and applications
4	489	24	2004	Radiology	T2 relaxation time of cartilage at MR imaging: comparison with severity of knee osteoarthritis
5	486	17	1996	Magnetic Resonance in Medicine	Gd-DTPA(2-) as a measure of cartilage degradation
6	423	60	2017	Radiographics	Shear-wave elastography: basic physics and musculoskeletal applications
7	418	20	2003	Radiology	Osteoarthritis: MR imaging findings in different stages of disease and correlation with clinical findings
8	406	15	1997	Radiology	Glycosaminoglycan in articular cartilage: in vivo assessment with delayed Gd(DTPA)(2-)-enhanced MR imaging
9	397	16	1999	Skeletal Radiology	MR imaging and CT in osteoarthritis of the lumbar facet joints
10	387	19	2004	Radiographics	From the archives of the AFIP - benign musculoskeletal lipomatous lesions
11	386	15	1999	Radiographics	Imaging of musculoskeletal neurogenic tumors: radiologic-pathologic correlation
12	384	11	1989	Radiology	Occult cartilage and bone injuries of the knee – detection, classification, and assessment with MR imaging
13	353	15	2001	Magnetic Resonance in Medicine	Proteoglycan-induced changes in T1ρ-relaxation of articular cartilage at 4T
14	335	13	1998	Radiology	MR imaging of the lumbar spine: prevalence of intervertebral disk extrusion and sequestration, nerve root compression, end plate abnormalities, and osteoarthritis of the facet joints in asymptomatic volunteers
15	298	17	2006	NMR in Biomedicine	Quantitative MRI of cartilage and bone: degenerative changes in osteoarthritis
16	296	25	2012	British Journal of Radiology	Ultrasound elastography for musculoskeletal applications
17	295	15	2004	Magnetic Resonance in Medicine	T2 and T1, MRI in articular cartilage systems
18	291	7	1983	Radiology	Musculoskeletal applications of nuclear magnetic resonance
19	282	47	2018	Radiology	Use of 2D U-Net convolutional neural networks for automated cartilage and meniscus segmentation of knee MR imaging data to determine relaxometry and morphometry
20	279	13	2002	Journal of Vascular and Interventional Radiology	Percutaneous vertebroplasty for osteoporotic compression fractures: quantitative prospective evaluation of long-term outcomes
21	278	13	2003	Skeletal Radiology	Comparison of fixed-flexion positioning with fluoroscopic semi-flexed positioning for quantifying radiographic joint-space width in the knee: test-retest reproducibility
22	276	13	2003	Radiographics	Radionuclide bone imaging: an illustrative review
23	275	11	2000	Investigative Radiology	Magic-angle effect in magnetic resonance imaging of articular cartilage - a review
24	274	9	1993	Skeletal Radiology	Benign bone-forming lesions – osteoma, osteoid osteoma, and osteoblastoma – clinical, imaging, pathological and differential considerations
25	270	16	2007	Ultrasound of the Musculoskeletal System	Ultrasound of the musculoskeletal system
26	269	8	1989	Radiology	Musculoskeletal neoplasms – static and dynamic GD-DTPA enhanced MR imaging
27	264	9	1994	American Journal of Roentgenology	Detection of soft-tissue hyperemia – value of power Doppler sonography
28	250	42	2018	Magnetic Resonance in Medicine	Super-resolution musculoskeletal MRI using deep learning
29	250	10	2000	Radiographics	Bone contusion patterns of the knee at MR imaging: footprint of the mechanism of injury
30	249	12	2003	IEEE Transactions on Medical Imaging	A robust method for registration of three-dimensional knee implant models to two-dimensional fluoroscopy images
31	248	6	1984	American Journal of Roentgenology	Magnetic resonance imaging of the normal and ischemic femoral head
32	246	8	1994	Radiology	Benign and malignant musculoskeletal lesions – dynamic contrast-enhanced MR imaging – parametric first pass images depict tissue vascularization and perfusion
33	245	9	1996	Radiology	Power Doppler sonography of synovitis: assessment of therapeutic response - preliminary observations
34	244	11	2002	Magnetic Resonance in Medicine	23Na MRI accurately measures fixed charge density in articular cartilage
35	235	24	2014	Radiographics	Fat-suppression techniques for 3-T MR imaging of the musculoskeletal system
36	235	12	2005	Radiographics	From the archives of the AFIP - imaging of musculoskeletal liposarcoma with radiologic-pathologic correlation
37	235	9	1998	American Journal of Roentgenology	Differentiation of necrotizing fasciitis and cellulitis using MR imaging
38	232	12	2005	Radiology	Triad of MR arthrographic findings in patients with cam-type femoroacetabular impingement
39	232	10	2001	Radiology	FDG PET of primary benign and malignant bone tumors: standardized uptake value in 52 lesions
40	231	39	2018	Magnetic Resonance in Medicine	Deep convolutional neural network and 3D deformable approach for tissue segmentation in musculoskeletal magnetic resonance imaging
41	224	15	2009	Radiology	Knee joint: comprehensive assessment with 3D isotropic resolution fast spin-echo MR imaging-diagnostic performance compared with that of conventional MR imaging at 3.0 T
42	219	15	2009	American Journal of Roentgenology	Ischiofemoral impingement syndrome: an entity with hip pain and abnormalities of the quadratus femoris muscle
43	215	24	2015	American Journal of Roentgenology	Sarcopenia: current concepts and imaging implications
44	213	7	1995	Radiographics	From the archives of the AFIP – musculoskeletal angiomatous lesions – radiologic-pathologic correlation
45	210	15	2010	American Journal of Neuroradiology	Lumbosacral transitional vertebrae: classification, imaging findings, and clinical relevance
46	210	12	2007	Journal of Magnetic Resonance Imaging	Magnetic resonance imaging with ultrashort TE (UTE) PULSE sequences: technical considerations
47	209	16	2011	Radiology	Identification of intraarticular and periarticular uric acid crystals with dual-energy CT: initial evaluation
48	208	42	2019	Medical Image Analysis	Automated segmentation of knee bone and cartilage combining statistical shape knowledge and convolutional neural networks: data from the Osteoarthritis Initiative
49	208	12	2007	Journal of Magnetic Resonance Imaging	Image-based musculoskeletal modeling: applications, advances, and future opportunities
50	208	11	2005	Radiology	Abductor tendons and muscles assessed at MR imaging after total hip arthroplasty in asymptomatic and symptomatic patients
51	208	6	1991	Radiology	Osteomyelitis – characteristics and pitfalls of diagnosis with MR imaging
52	202	16	2011	Radiology	Cartilage in anterior cruciate ligament-reconstructed knees: MR imaging T1ρ and T2 – initial experience with 1-year follow-up
53	200	14	2010	Radiographics	MR imaging of patellar instability: injury patterns and assessment of risk factors
54	199	8	2000	Radiographics	Imaging of extrapulmonary tuberculosis
55	198	15	2011	Magnetic Resonance Imaging	Quantitative MRI using T1ρ, and T2 in human osteoarthritic cartilage specimens: correlation with biochemical measurements and histology
56	198	13	2009	Radiology	Soft-tissue tumors and tumor-like lesions: a systematic imaging approach
57	197	11	2006	American Journal of Roentgenology	Association between rotator cuff abnormalities and reduced acromiohumeral distance
58	196	22	2015	Journal of Magnetic Resonance Imaging	UTE imaging in the musculoskeletal system
59	195	12	2008	Radiology	Cortical bone water: in vivo quantification with ultrashort echo-time MR imaging
60	195	8	2000	Investigative Radiology	MRI techniques in early stages of cartilage disease
61	194	13	2009	European Radiology	T1rho, T2 and focal knee cartilage abnormalities in physically active and sedentary healthy subjects versus early OA patients – a 3.0-Tesla MRI study
62	192	16	2012	Journal of Magnetic Resonance Imaging	Does vertebral bone marrow fat content correlate with abdominal adipose tissue, lumbar spine bone mineral density, and blood biomarkers in women with type 2 diabetes mellitus?
63	188	12	2008	American Journal of Roentgenology	The top 10 reasons musculoskeletal sonography is an important complementary or alternative technique to MRI
64	188	8	2001	Skeletal Radiology	Gender differences in knee joint cartilage thickness, volume and articular surface areas: assessment with quantitative three-dimensional MR imaging
65	188	6	1993	Radiology	Diagnosis of osteomyelitis – utility of fat-suppressed contrast-enhanced MR imaging
66	188	6	1992	American Journal of Roentgenology	The scintigraphic diagnosis of osteomyelitis
67	186	14	2011	Skeletal Radiology	Diffusion-weighted imaging (DWI) in musculoskeletal MRI: a critical review
68	186	8	2001	Seminars in Ultrasound CT and MRI	Real-time spatial compound imaging: application to breast, vascular, and musculoskeletal ultrasound
69	185	15	2012	European Radiology	Metal artefact reduction in gemstone spectral imaging dual-energy CT with and without metal artefact reduction software
70	185	8	2002	British Journal of Radiology	Skeletal aspects of Gaucher disease: a review
71	184	12	2008	Radiology	Bone and soft-tissue lesions: what factors affect diagnostic yield of image-guided core-needle biopsy?
72	183	9	2003	Skeletal Radiology	SAPHO: syndrome or concept? Imaging findings
73	181	6	1994	Radiology	Gender differences in vertebral sizes in adults – biomechanic implications
74	180	10	2006	NMR in Biomedicine	Quantitative MRI for the assessment of bone structure and function
75	178	30	2018	Radiology	Deep learning approach for evaluating knee MR images: achieving high diagnostic performance for cartilage lesion detection
76	178	9	2005	Radiographics	Hamstring muscle complex: an imaging review
77	178	7	1998	American Journal of Roentgenology	Accuracy of CT-guided needle biopsy of musculoskeletal neoplasms
78	177	22	2016	Radiology	Dual-energy CT for the musculoskeletal system
79	177	12	2009	Radiology	MR imaging insights into skeletal maturation: what is normal?
80	176	22	2016	Quantitative Imaging in Medicine and Surgery	The imaging of osteomyelitis
81	174	11	2008	IEEE Transactions on Medical Imaging	A technique for regional analysis of femorotibial cartilage thickness based on quantitative magnetic resonance imaging
82	173	8	2002	Skeletal Radiology	Accuracy of CT-guided biopsies in 359 patients with musculoskeletal lesions
83	173	5	1987	Radiology	Primary musculoskeletal tumors – examination with MR imaging compared with conventional modalities
84	172	11	2008	Skeletal Radiology	MR observations of long-term musculotendon remodeling following a hamstring strain injury
85	172	5	1992	Radiology	Diagnosis of soft-tissue masses with MR imaging – can benign masses be differentiated from malignant ones
86	169	27	2014	Radiology	Dedicated cone-beam CT system for extremity imaging
87	169	11	2008	Magnetic Resonance in Medicine	In vivo T1ρ mapping in cartilage using 3D magnetization-prepared angle-modulated partitioned k-space spoiled gradient echo snapshots (3D MAPSS)
88	168	7	1999	Journal of Nuclear Medicine	Evaluation of neoadjuvant therapy response of osteogenic sarcoma using FDG PET
89	166	7	2000	European Radiology	Carpal tunnel syndrome: usefulness of sonography
90	163	6	1998	Radiology	Chronic osteomyelitis: detection with FDG PET and correlation with histopathologic findings
91	162	8	2004	Radiology	Proteoglycan loss in human knee cartilage: quantitation with sodium MR imaging - feasibility study
92	162	6	1998	Radiology	Cancellous bone volume and structure in the forearm: noninvasive assessment with MR microimaging and image processing
93	162	5	1993	Radiology	Enhancement of joint fluid with intravenously administered gadopentetate dimeglumine – technique, rationale, and implications
94	160	7	2001	Radiographics	Imaging of musculoskeletal fibromatosis
95	159	11	2009	Seminars in Nuclear Medicine	Nuclear medicine and the infected joint replacement
96	157	13	2012	Radiology	Musculoskeletal tumors: how to use anatomic, functional, and metabolic MR techniques
97	157	9	2006	American Journal of Roentgenology	Sensitivity of MR arthrography in the evaluation of acetabular labral tears
98	156	13	2012	Radiology	Factors associated with meniscal extrusion in knees with or at risk for osteoarthritis: the multicenter osteoarthritis study
99	155	13	2012	European Radiology	Clinical indications for musculoskeletal ultrasound: a Delphi-based consensus paper of the European Society of Musculoskeletal Radiology
100	149	11	2010	Seminars in Nuclear Medicine	PET and SPECT in osteomyelitis and prosthetic bone and joint infections: a systematic review

Year of Publication

**Figure 1:**
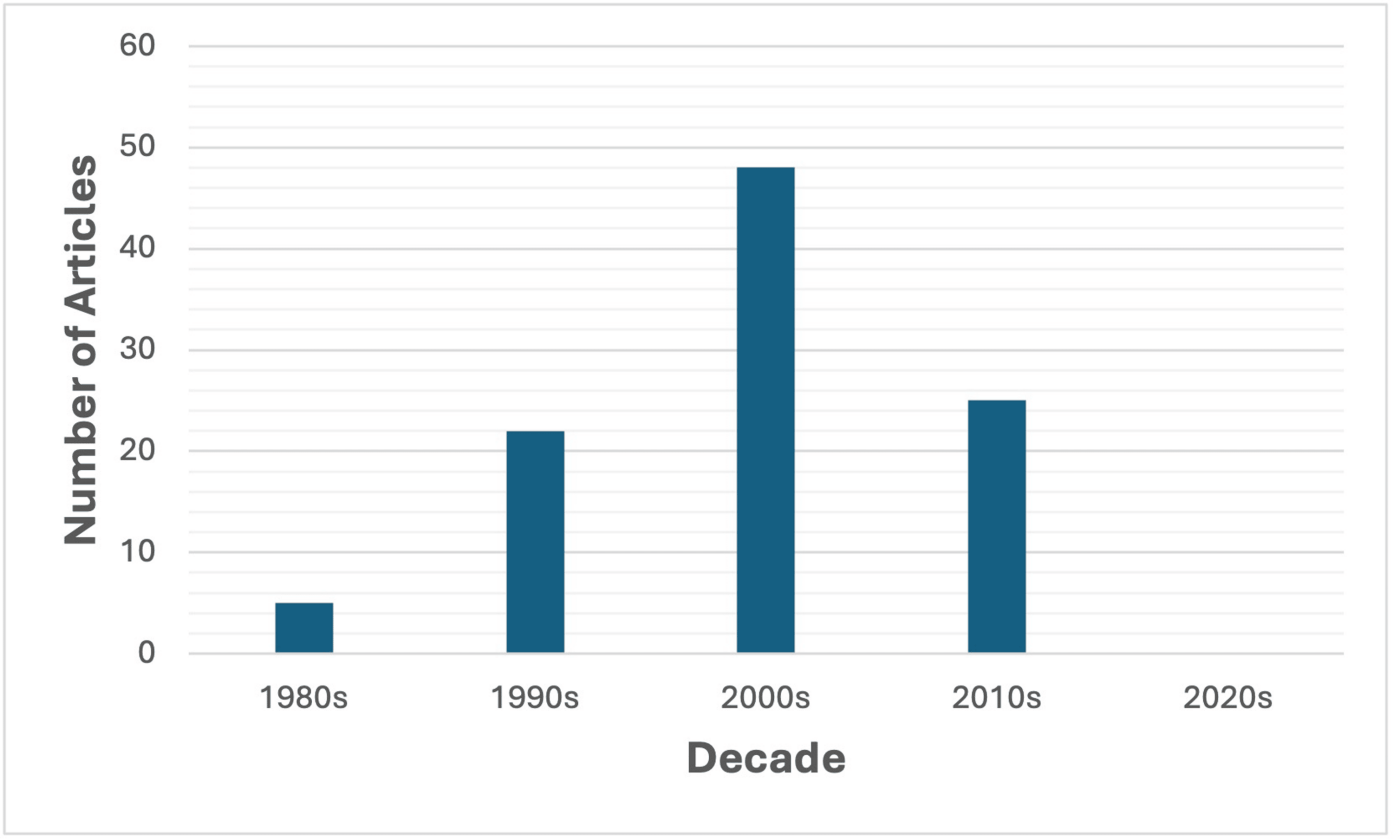
Distribution of the top 100 most cited articles by decade of publication

The top 100 most cited articles were published between 1983 and 2019. 2008, 2009, and 2012 had the greatest number of publications, with six articles each year. The decade with the highest number of top cited articles was the 2000s with 48 articles, followed by the 2010s with 22 articles (Figure 1). The earliest article in the top 100 list is from 1983, published in Radiology, entitled "Musculoskeletal applications of nuclear magnetic resonance" [9]. Twelve of the top 100 articles were published in the last decade (2014 or after). 

Journals

The top 100 articles were published across 21 journals. The journal with the most articles in the top 100 list was Radiology with a contribution of 34 publications. At the time of writing, Radiology was the journal with the highest IF (IF: 12.1). This was followed by Radiographics (IF: 5.2) which contributed 12 articles and the American Journal of Roentgenology (IF: 4.7) which contributed 11 articles. Magnetic Resonance Imaging (IF: 2.1) contributed eight. Two journals contributed four articles each and five journals contributed two articles to the list. Nine journals contributed one article each to the list. 

Country of Origin 

**Figure 2:**
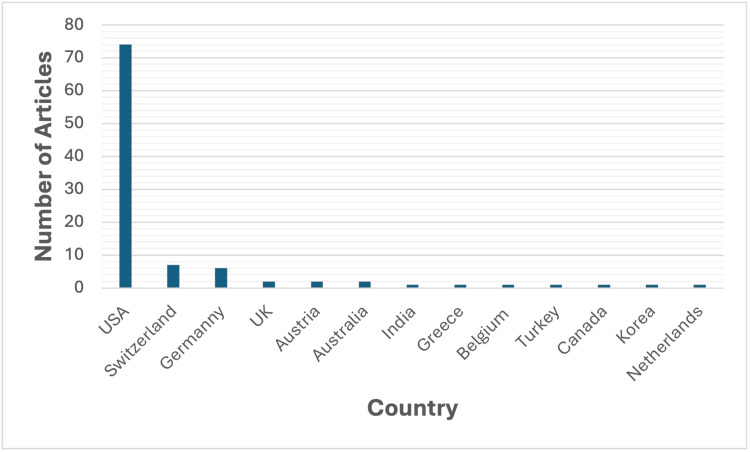
Distribution of articles by country of publication USA: United States of America; UK: United Kingdom

Figure 2 shows the distribution of articles by country of publication. The United States (USA) had the greatest number of publications on the list (n=74), followed by Switzerland (n=7). 

Authors and Institution of Origin 

In total, there were 87 first authors on the top 100 list. Nine authors were the first authors of more than one article. Overall, 65 different institutions contributed articles to the top 100 list. The University of California produced the highest number of articles (n=11), followed by the University of Pennsylvania (n=6), Orthopedic University Clinic Balgrist (n=4), and Massachusetts General Hospital (n=4). Beth Israel Deaconess Medical Center and the Armed Forces Institute of Pathology published three articles each. 

Modality

Articles were distributed across a range of radiological modalities, as seen in Table 2. 

**Table 2:** Distribution of articles by modality *22 articles focused on more than one modality MRI: magnetic resonance imaging; CT: computed tomography; US: ultrasound; XR: X-ray; PET: positron emission tomography; DEXA: dual-energy X-ray absorptiometry

	Descriptor	Frequency (n)
Modality*	MRI	73
	CT	18
	US	14
	XR	10
	PET	6
	Interventional	4
	Scintigraphy	3
	DEXA	2
	Artificial intelligence	1
	Fluoroscopy	1

Discussion

MSDs are a leading cause of disability worldwide, second only to substance use and mental health disorders, with major consequences for the overall health of the global population [10]. They are prevalent across all ages and can have an immense societal cost in the form of chronic pain, depression, limitations in the ability to work and to participate in social roles, early retirement, and increased risk of development of other chronic diseases [11]. This article gives an interesting insight into the many journals, institutions, and countries that have contributed most to the field of MSK radiology and how the subject matter and areas of interest have developed over time. 

The most cited article in this field is "Femoroacetabular impingement: radiographic diagnosis - what the radiologist should know" which was published in the American Journal of Roentgenology in 2007 [7]. This article has, at the time of conducting this analysis, received 709 citations. Since its publication in 2007, it has been cited an average of 42 times per year, indicating its influential impact on MSK radiology. 

It is widely accepted that high citation counts correlate with a greater impact in the research field [12]. Likewise, the IF of a journal is traditionally seen as a crude yet still important approximation of the esteem and influence of a journal. It is calculated by dividing the number of article citations that the journal received by the number of articles published in the previous two years [12]. However, citation metrics are essentially dependent on the assumption that a simple linear relationship exists between scientific quality and citation counts, when, in fact, many factors may compound this relationship [12]. The impact of a piece of research could also be considered to be the degree to which it has been useful to other researchers and its effect on clinical practice [12], for example, the impact it has on clinicians who may not publish on a regular basis but do use literature to inform their practice. These metrics are not accounted for when citation count alone is used to measure an article's impact on a particular field [12,13]. Furthermore, an article's perceived impact, in relation to citation count, may also be affected by factors such as the size of the chosen subject field, the location of the author, journal IF, and published language [12-14]. Another limitation is that it can take months, even years, for an article to accumulate citations [13]. 

As a complement to these traditional measures of scholarly impact, a new area known as altmetrics has become a subject of interest [14]. Altmetrics allow us to measure and monitor online attention markers related to published research in order to see where research is having an impact. These markers facilitate analysis of "hidden" indicators of impact including download counts, social media shares, mentions, and numbers of followers. Initial metrics can also be analyzed with artificial intelligence (AI) to predict the scholarly impact of research at an early stage which may accelerate the overall progress of research [14]. 

The majority of studies (74%) originated from the United States. This is similar to research output in other fields of radiology [3,15,16]. This highlights the United States' significant role in terms of scientific research output. This may be partly attributable to the large size of the United States' radiologic community. It has also been noted that US authors may be more inclined to cite authors from other US institutions, thus increasing their representation in the literature [17,18]. 

While MSK radiology may have had its roots in plain film, our paper highlights how imaging modalities utilized in MSK radiology have evolved over time. The introduction of new imaging modalities has facilitated the diagnosis and treatment of conditions that were previously out of reach when using plain film alone, thus providing ample opportunities for diagnostic and therapeutic advancement in MSK radiology [19]. As evident in the top 100 list, cross-sectional and functional imaging are playing ever-increasing roles in MSK diagnostics. Interventional radiology (IR) techniques are being performed using a range of modalities, including computed tomography (CT), ultrasound (US), and magnetic resonance imaging (MRI). IR plays an important role in MSK tumor and infection diagnosis and also the treatment of joint disease [19].

MRI is the most common imaging modality represented in the top 100 list (n=73). The earliest article in our list, published in 1983 by Moon and colleagues, pertains to MRI, entitled "Musculoskeletal applications of nuclear magnetic resonance" [9]. Over the years, as MR image quality improved and imaging time decreased, the number of articles published relating to MRI notably increased, as is evident from the list of the top 100 most cited articles. 

CT is the modality of interest in 18 articles, the earliest of which was published in 1995. The most recent paper pertaining to CT was published in 2016, "Dual-energy CT for the musculoskeletal system" by Mallinson et al. [20]. There have been many recent advancements in CT imaging with a wealth of MSK applications mentioned on the top 100 list, including dual-energy CT with metal artifact reduction software and also cone-beam CT, which utilizes flat-panel detectors that can be used to image the lower extremities while weight-bearing [20,21]. Not mentioned is four-dimensional CT, which captures a structure in motion and can lend new insights into phenomena such as joint kinematics, instability, and impingement, or, most recently, photon-counting CT with metal artifact reduction [22,23].

Eighteen articles in the top 100 list compared the diagnostic ability of one modality against the other. Often, one imaging modality alone may not be sufficient in the definitive diagnosis of MSK diseases [24]. For this reason, multimodality imaging systems that overcome the limitations of individual systems have been developed. In particular, hybrid positron emission tomography (PET)-MRI systems are an emerging technology worthy of further research and are notably absent from the top 100 list. This modality incorporates MRI's precise soft tissue detail with the essential molecular information on early metabolic changes from PET [25]. While its use for imaging osteosarcoma and some MSK infections has been established, its widespread application is still limited, partly due to the limited availability of systems, challenges in workflow, and issues with ionizing radiation doses, particularly in chronic diseases [25]. The challenge for its widespread adoption into clinical practice remains, and future research may pave the way.

US was the primary modality in 14 articles. US stands as one of the most rapidly expanding imaging techniques in MSK radiology [26]. For example, elastography is a US modality that is increasingly being incorporated into MSK imaging. It has many advantages over other methods of elasticity estimation, such as MR elastography [26]. US is low-cost, fast, and noninvasive and has the potential for wider clinical availability compared to other modalities [27]. Its applications are numerous and include assessment of vascularity, e.g., with MSK tumors, or complex anatomical structures such as the rotator cuff [27]. Guided interventions are among the most rapidly growing applications of MSK US. Due to the potential for clear sonographic visualization of needles, the real-time visualization of injected or aspirated material, and the excellent near-field resolution that it can afford, it is likely that US will continue to grow in use in relation to MSK radiology [27].

Only one article out of the top 100 list pertains to AI, entitled "Use of 2D U-Net convolutional neural networks for automated cartilage and meniscus segmentation of knee MR imaging data to determine relaxometry and morphometry" published in Radiology in 2018 [28]. AI is increasingly being used in radiology to increase the workflow, decrease radiological errors, and improve patient care [29]. This is especially pertinent in a landscape where the global burden of MSK disorders and demand for diagnostic radiology is increasing at a rate that outweighs the number of available radiologists [30]. AI has many applications in MSK radiology already available, from using deep learning to improve sensitivity in identifying ligament tears and fractures to classification algorithms for osteoarthritis [29]. It is evident that there is potential for AI to enhance many other aspects of MSK radiology, such as the evaluation of orthopedic implant position and potential postoperative complications such as peri-prosthetic infection and component wear [29]. Research has also demonstrated the potential for AI to aid in the diagnosis of bone and soft tissue tumors [29]. There are AI programs currently commercially available, including algorithms for the estimation of bone age and the detection of fractures [29]. However, one of the main limitations of the widespread incorporation of AI into clinical practice is the relative paucity of external, independent validation of AI programs [30]. Thus, continued multicenter collaboration will be imperative for this burgeoning field of research. 

This study has limitations. The use of citation count is a source of bias; the more time that has passed since the publication of an article, the more likely it is to be cited over time, and therefore, it is more likely to have a greater number of citations. To reduce this potential source of bias, the average citations per year were also included. Some articles may have been inadvertently excluded as a result of the search criteria used. In addition, using journal IF from one particular year (2024) does not allow for temporal fluctuations in IF over time. Furthermore, the potential bias of self-citation was not accounted for in this study. This article is not an exhaustive list of the top 100 publications in the field of MSK radiology; however, it does serve to highlight some of the most influential papers to date.

## Conclusions

Over the past 40 years, significant advances have been made in MSK radiology. This study provides a detailed analysis of the top 100 most cited articles in this radiological subspecialty. It highlights the most influential papers in MSK radiology over time, the characteristics of these studies, as well as the potential future trends in MSK radiology research, such as the use of AI in order to maximize radiologist workflow efficiency and to improve patient outcomes. 
